# Transactions of the Michigan State Medical Society

**Published:** 1882-09-20

**Authors:** 


TRANSACTIONS OF THE MICHIGAN STATE MEDICAL SOCIETY, for the year 1882, No. 11, Vol. viij.

Kindly sent us by Geo. E. Ranney, M. D., Recording Secretary, of Lansing, Mich. The society assembled at Ypsilanti, May 10, 1882. The address of welcome was made by Capt. E. P. Allen. The annual address, by the President, Dr. J. H. Jerome, and interesting papers presented by the following gentlemen:

Dr. Christian—Mal presentation.
Dr. Burr—Insanity of Masturbation.
Dr. Pratt—Legal Responsibility of Surgeons.
Dr. Shurley—Laryngeal Phthisis.
Dr. Reynolds—Treatment of Measles and Scarlet Fever.
Dr. E. Smith—Suppurative Catarrh of Middle Ear.
Dr. Conner—Optic Neuritis.
Dr. H. J. Reynolds—After Treatment of Tracheotomy.
Dr. Wade—Antiseptics in Treatment of Disease.
Dr. E. B. Ward—Æsthetics in Medicine.
Dr. Post—Urinary Deposits, etc.
Dr. Ranney—Notes on Uterine Tumors.

The volume contains 276 oc. pages, and is creditably gotten up. The papers are interesting, practical in character, and some of them written with marked ability.

President elect for the ensuing year, Dr. G. W. Topping, Detroit.

Secretary, Dr. Geo. E. Ranney, of Lansing.

The Society will meet next at Kalamazoo, at such time as the President and Secretary shall hereafter appoint.

